# Supplementary material for: Dental microwear texture analysis reveals behavioural, ecological and habitat signals in Late Jurassic sauropod dinosaur faunas
Source: Nat Ecol Evol. 2025 Jul 18;9(9):1719–30. doi: 10.1038/s41559-025-02794-5 (PMC12420387; doi:10.1038/s41559-025-02794-5)
Supplement: Supplementary file 1 — Supplementary Fig. 1. [file 41559_2025_2794_MOESM1_ESM.pdf]

nature ecology & evolution

Supplementary information

https://doi.org/10.1038/s41559-025-02794-5

Dental microwear texture analysis reveals behavioural, ecological and habitat signals in Late Jurassic sauropod dinosaur faunas

In the format provided by the authors and unedited

Supplemental Material for

Dental microwear texture analysis reveals behavioural, ecological, and habitat signals in Late Jurassic sauropod dinosaur faunas

Table of content:

Supplementary Figure 1. Boxplots for all 25 dental microwear texture analysis parameters, according to clade, for buccal and occlusal surfaces, compared between the three sauropod faunas. The thick horizontal bar represents the median; the box encloses the first (25%) and third (75%) quartiles; the whiskers extend to the full interquartile range Parameters are sorted according to functional category. Number of surfaces per taxon with number of specimens (in case buccal and occlusal surface of the same specimen were included) given in parenthesis, Turiasauria: n = 5(7), Titanosauriformes: n = 9(15), Camarasauridae: n = 12(20), Flagellicaudata: n = 12(13), Macronaria indet: n = 3(4), sauropoda indet: n = 1(1). Brachiosauridae = pink, Camarasauridae = orange, Flagellicaudata = purple, Turiasauria = blue, Mamenchisauridae = dark grey, unidentified macronarians = yellow. Parameter descriptions are given in Supplementary Table 6.

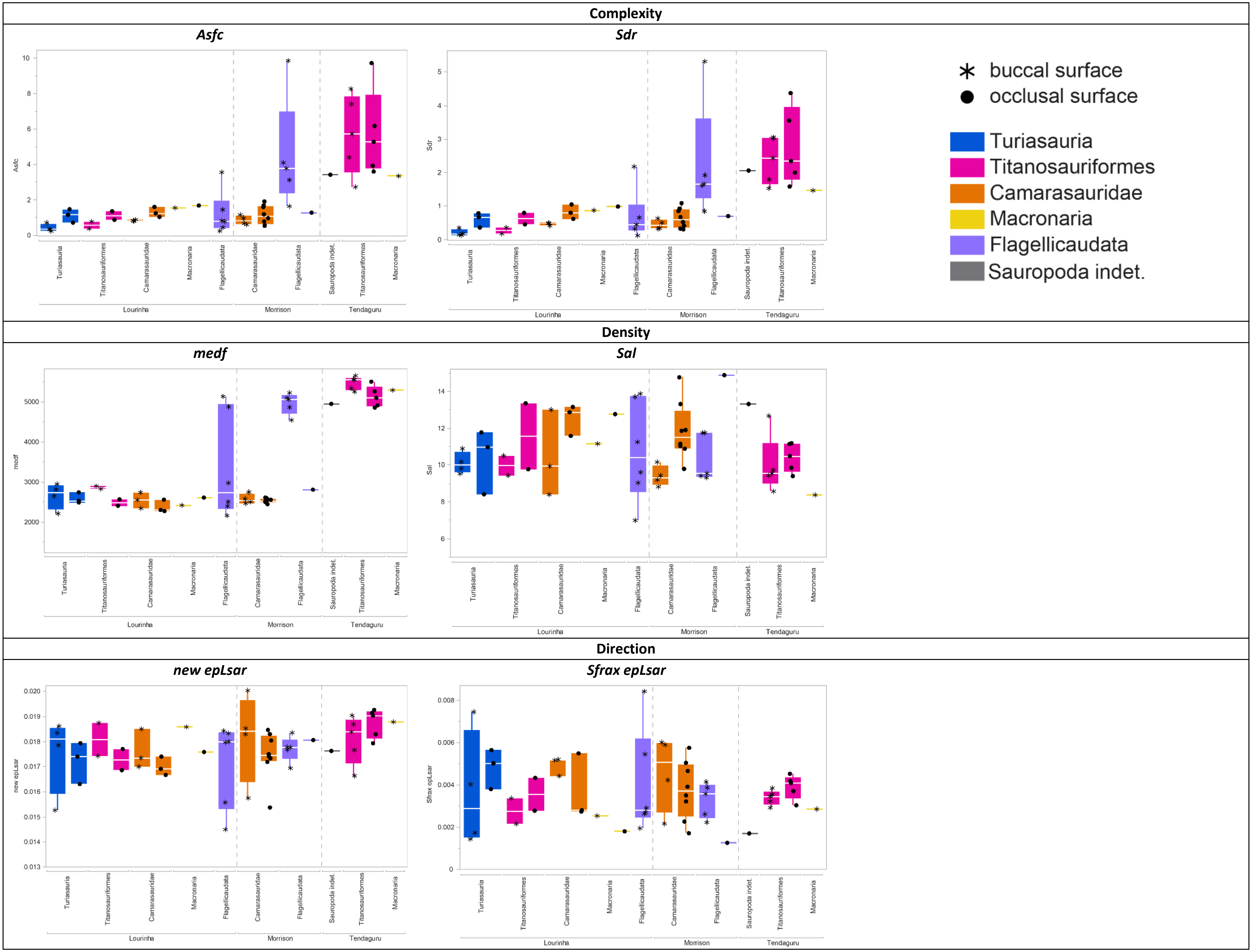

Complexity
Asfc
Sdr
Density
medf
Sal
Direction
new epLsar
Sfrax epLsar
buccal surface
occlusal surface
Turiasauria
Titanosauriformes
Camarasauridae
Macronaria
Flagellicaudata
Sauropoda indet.
Lourinha
Morrison
Tendaguru

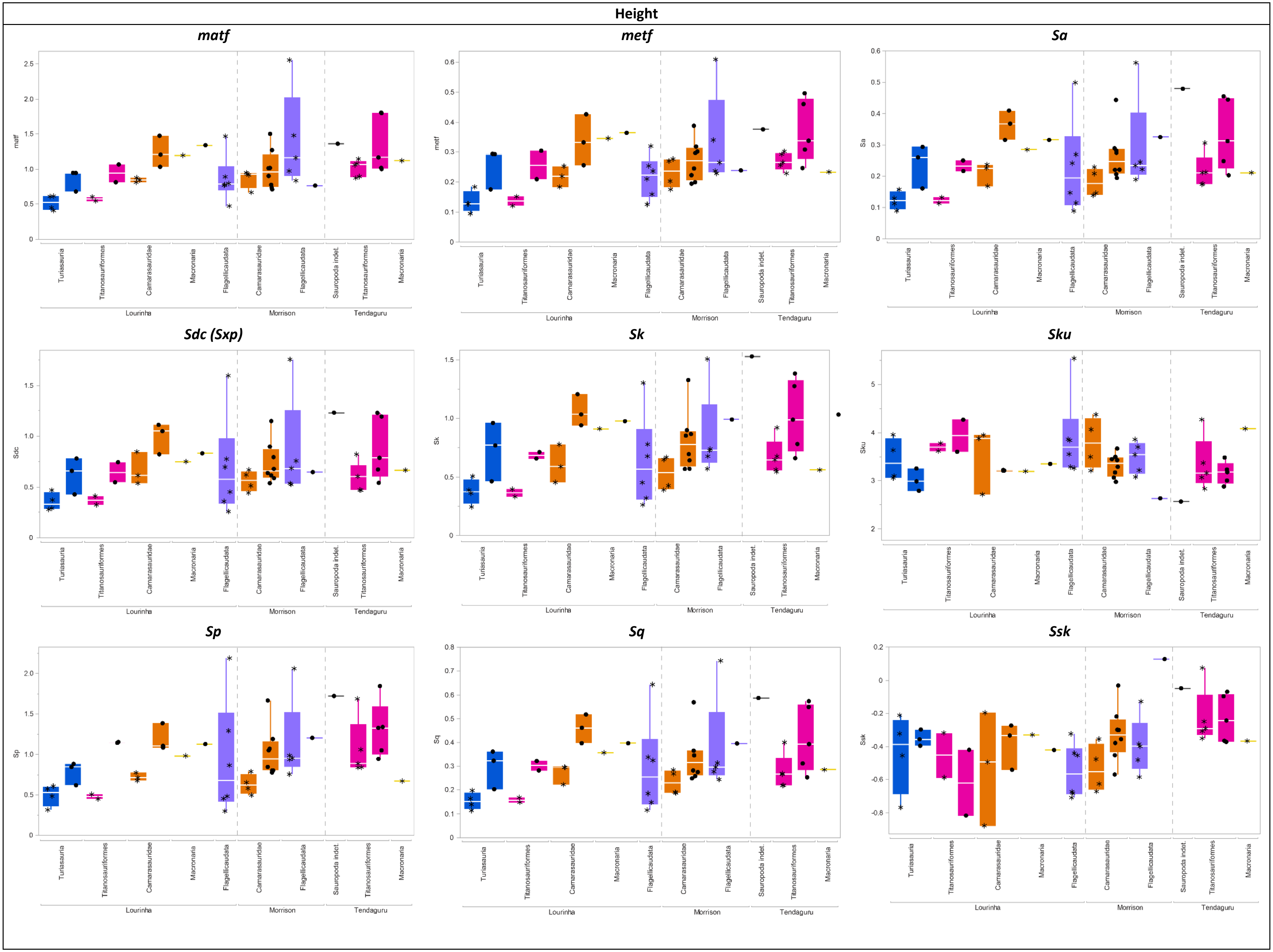
Height
matf
metf
Sa
Sdc (Sxp)
Sk
Sku
Sp
Sq
Ssk
Turiasauria
Titanosauriformes
Camarasauridae
Macronaria
Flagellicaudata
Sauropoda indet.
Lourinha
Morrison
Tendaguru

Height (continued)

Sv

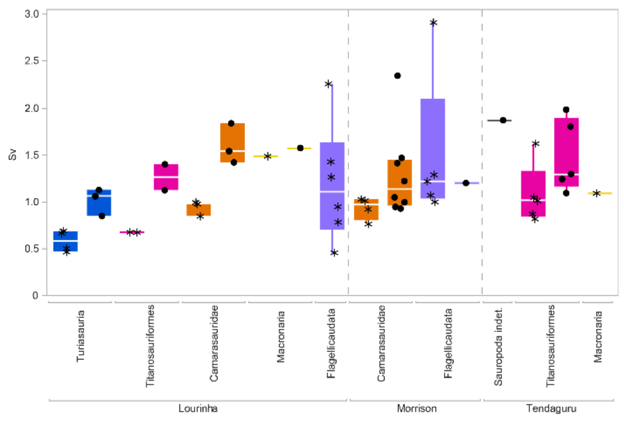

Sz

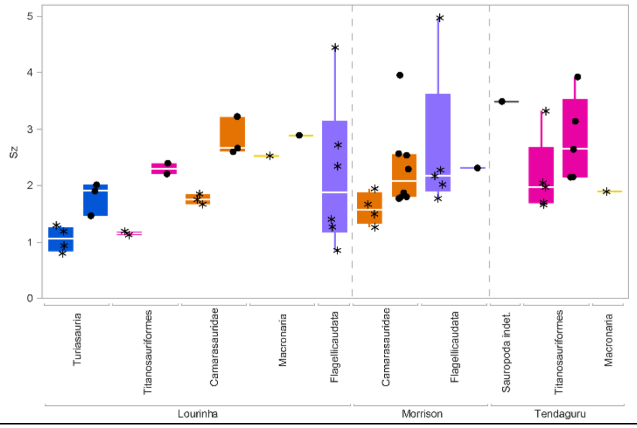

Plateau size

Smc

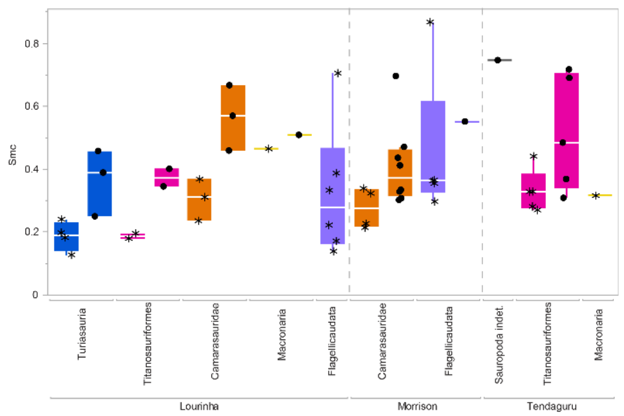

Smr

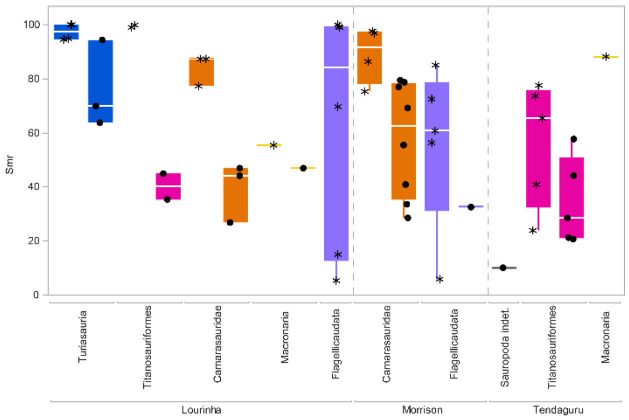

Slope

Sdq

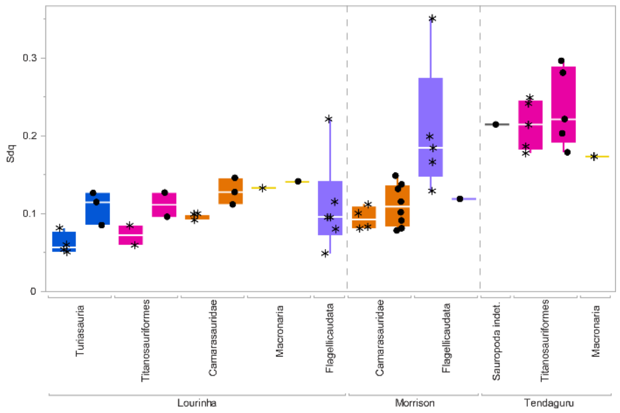

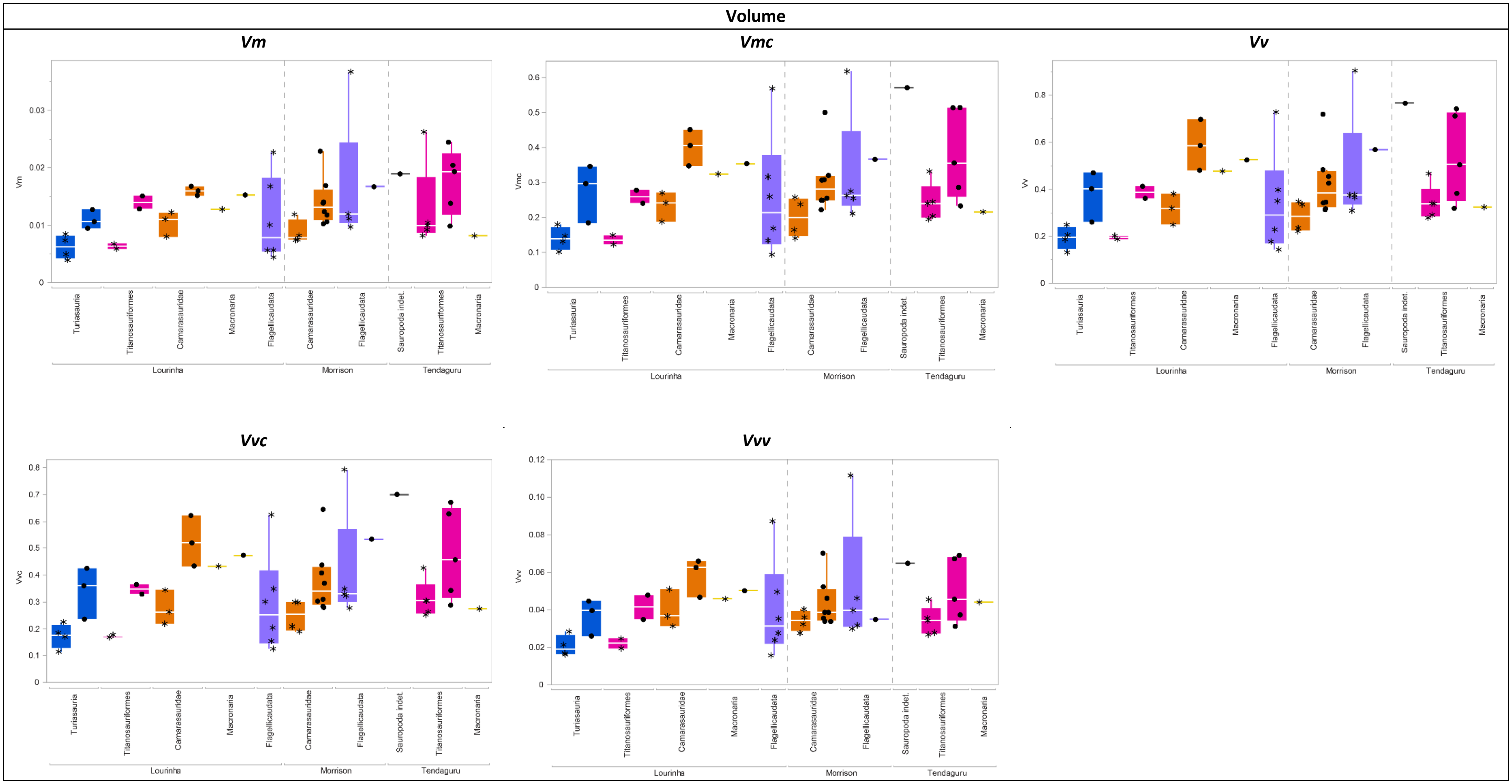
Volume
Vm
Vmc
Vv
Vvc
Vvv
Vm
Vmc
Vv
Vvc
Vvv
0
0.01
0.02
0.03
0.1
0.2
0.3
0.4
0.5
0.6
0.7
0.8
0.02
0.04
0.06
0.08
0.10
0.12
Turiasauria
Titanosauriformes
Camarasauridae
Macronaria
Flagellicaudata
Camarasauridae
Flagellicaudata
Sauropoda indet.
Titanosauriformes
Macronaria
Lourinha
Morrison
Tendaguru
