# Supplementary material for: Dental microwear texture analysis reveals behavioural, ecological and habitat signals in Late Jurassic sauropod dinosaur faunas
Source: Nat Ecol Evol. 2025 Jul 18;9(9):1719–30. doi: 10.1038/s41559-025-02794-5 (PMC12420387; doi:10.1038/s41559-025-02794-5)
Supplement: Supplementary file 2 — Reporting Summary [file 41559_2025_2794_MOESM2_ESM.pdf]

nature portfolio

Corresponding author(s): Daniela E. WInkler, Emanuel Tschopp

Last updated by author(s): May 23, 2025

Reporting Summary

Nature Portfolio wishes to improve the reproducibility of the work that we publish. This form provides structure for consistency and transparency in reporting. For further information on Nature Portfolio policies, see our Editorial Policies and the Editorial Policy Checklist.

Statistics

For all statistical analyses, confirm that the following items are present in the figure legend, table legend, main text, or Methods section.

n/a	Confirmed	
☐	☒	The exact sample size (*n*) for each experimental group/condition, given as a discrete number and unit of measurement
☐	☒	A statement on whether measurements were taken from distinct samples or whether the same sample was measured repeatedly
☐	☒	The statistical test(s) used AND whether they are one- or two-sided<br>*Only common tests should be described solely by name; describe more complex techniques in the Methods section.*
☒	☐	A description of all covariates tested
☐	☒	A description of any assumptions or corrections, such as tests of normality and adjustment for multiple comparisons
☐	☒	A full description of the statistical parameters including central tendency (e.g. means) or other basic estimates (e.g. regression coefficient) AND variation (e.g. standard deviation) or associated estimates of uncertainty (e.g. confidence intervals)
☒	☐	For null hypothesis testing, the test statistic (e.g. *F*, *t*, *r*) with confidence intervals, effect sizes, degrees of freedom and *P* value noted<br>*Give P values as exact values whenever suitable.*
☒	☐	For Bayesian analysis, information on the choice of priors and Markov chain Monte Carlo settings
☒	☐	For hierarchical and complex designs, identification of the appropriate level for tests and full reporting of outcomes
☒	☐	Estimates of effect sizes (e.g. Cohen's *d*, Pearson's *r*), indicating how they were calculated

Our web collection on statistics for biologists contains articles on many of the points above.

Software and code

Policy information about availability of computer code

Data collection	Sensofar optical profiler equipped with software SensoSCAN v.6.7
Data analysis	DMTA was conducted in MountainsMap v.9.1, Besancon, France; statistics were conducted in JMP pro v.17

For manuscripts utilizing custom algorithms or software that are central to the research but not yet described in published literature, software must be made available to editors and reviewers. We strongly encourage code deposition in a community repository (e.g. GitHub). See the Nature Portfolio guidelines for submitting code & software for further information.

Data

Policy information about availability of data

All manuscripts must include a data availability statement. This statement should provide the following information, where applicable:

- Accession codes, unique identifiers, or web links for publicly available datasets
- A description of any restrictions on data availability
- For clinical datasets or third party data, please ensure that the statement adheres to our policy

All measurements used for the analyses herein are included in the supplementary materials. Original scan files, based on which the measurements were taken are available on the UHH Forschungsdatenbank (https://www.fdr.uni-hamburg.de/) at https://doi.org/10.25592/uhhfdm.16175

Research involving human participants, their data, or biological material

Policy information about studies with human participants or human data. See also policy information about sex, gender (identity/presentation), and sexual orientation and race, ethnicity and racism.

Reporting on sex and gender	no human participants in this study
Reporting on race, ethnicity, or other socially relevant groupings	n/a
Population characteristics	n/a
Recruitment	n/a
Ethics oversight	n/a

Note that full information on the approval of the study protocol must also be provided in the manuscript.

Field-specific reporting

Please select the one below that is the best fit for your research. If you are not sure, read the appropriate sections before making your selection.

☐ Life sciences ☐ Behavioural & social sciences ☒ Ecological, evolutionary & environmental sciences

For a reference copy of the document with all sections, see nature.com/documents/nr-reporting-summary-flat.pdf

Ecological, evolutionary & environmental sciences study design

All studies must disclose on these points even when the disclosure is negative.

Study description	Analysis of mechanical dental wear features for diet inferences (dental microwear texture analysis) of sauropods from three different faunas was conducted to infer niche partitioning, competition, and potential migration patterns.
Research sample	The sample consists of sauropod teeth housed in different museum collections in the USA, Portugal, and Germany: in total it includes 39 sauropod individuals, 17 of which were recovered from the Lourinha Formation (Portugal), 13 from the Morrison Formation (USA), and nine from the Tendaguru Formation (Tanzania, curated at the Berlin Museum of Natural History)
Sampling strategy	When available, we measured wear texture directly on the original teeth, after cleaning the enamel surfaces with acetone and/or ethanol. If not available for loan, tooth specimens were moulded with high resolution dental silicone (Provil novo Light C.D.2 fast set EN ISO 4823, type 3 light, Heraeus Kulzer GmbH, Dormagen, Germany).
Data collection	Surface scanning was conducted at the Leibniz Institute for the Analysis of Biodiversity Change (LIB) in Hamburg, Germany.<br>We used the 3D profiling microscope Sensofar S neox (Sensofar-Tech, SL, Terrassa, Barcelona, Spain), with a blue LED (460 nm), vertical resolution of 0.07 µm, spatial resolution of 0.14 µm (in x, y) and numerical aperture of 0.9. The CCD camera resolution was 1232 x 1028 pixels, and the resulting scan size using a 100x objective was 175.44 x 132.10 µm. Each scan was manually cropped to 100 x 100 µm to exclude edge effects and damages present in several scans.
Timing and spatial scale	Surface scanning was conducted between June 2022 and November 2023 by AS and RW.
Data exclusions	All resulting visual data was put through two manual quality screenings, first done by AS, and then followed by DW. This assessment was done in order to exclude any surface scan and correlating measurements that still remained impacted by foreign structures. These features included sediment particles, along with consolidant and glue residues, and fractures of the enamel surface. Scans from molds and casts were checked for extra features, such as bubbles that formed during the molding and casting process. Furthermore, some scan surfaces included significant portions with plateau-like structures resulting from the digital infilling of holes during the data post-processing step. These scans were also deemed unusable for subsequent statistical analysis, because the large, completely flat areas would have significantly impacted overall texture quantification. As a result of the manual screening, each scan was attributed a value representative of its quality. The three levels created to indicate the scan quality are as follows: quality 1, good; quality 2, fair; and quality 3, bad. A total of 620 scans were deemed to be of good quality, whereas 27 were considered to be of fair quality. We identified 364 scans of bad quality, which should be excluded from future analysis. The majority of good scans resulted from 431 measurements taken from original teeth, followed by 182 taken from molds, and only 7 scans resulting from casts. Scans deemed of fair quality included 8 from original teeth, and 19 resulting from molds, with no scan from a cast falling within this quality level.
Reproducibility	Dental microwear texture analysis can be repeated using the same specimens, but re-scanning exactly the same surface area is hardly possible, as teeth or moulds are manually oriented on the microscope plate under the objective. As the measurement process and data analysis are automated, and all scripts for data treatment (filtering) are available, the repeatability is still higher than in 2D microwear studies.

Randomization	Groups were created according to taxonomic affinity
Blinding	Data analysis was done by DEW on specimens only identified by their inventory number, not with full taxonomic information.

Did the study involve field work? ☐ Yes ☒ No

Reporting for specific materials, systems and methods

We require information from authors about some types of materials, experimental systems and methods used in many studies. Here, indicate whether each material, system or method listed is relevant to your study. If you are not sure if a list item applies to your research, read the appropriate section before selecting a response.

Materials & experimental systems

n/a	Involved in the study
☒	☐ Antibodies
☒	☐ Eukaryotic cell lines
☐	☒ Palaeontology and archaeology
☒	☐ Animals and other organisms
☒	☐ Clinical data
☒	☐ Dual use research of concern
☒	☐ Plants

Methods

n/a	Involved in the study
☒	☐ ChIP-seq
☒	☐ Flow cytometry
☒	☐ MRI-based neuroimaging

Palaeontology and Archaeology

Specimen provenance	All material included in this study is already curated in the following museums:<br>AMNH FARB: American Museum of Natural History; Fossil Amphibian, Reptile, and Bird Collection, New York City, USA<br>CM: Carnegie Museum of Natural History, Pittsburgh, USA<br>CMC: Cincinnati Museum Center, Museum of Natural History & Science, Cincinnati, USA<br>LIB: Leibniz-Institut zur Analyse des Biodiversitätswandels, Bonn and Hamburg, Germany<br>MB.R.: Museum für Naturkunde, Berlin, Germany<br>ML: Museum of Lourinhã, Portugal<br>NMZ: Natural History Museum of the University of Zurich, Switzerland<br>SMA: Sauriermuseum Aathal, Switzerland<br>USNM: Smithsonian National Museum of Natural History, Washington DC, USA<br>YPM: Yale Peabody Museum, New Haven, USA<br>We therefore obtained permisson to include the samples into our analysis from the curators.
Specimen deposition	see museums listed above
Dating methods	no new dates were calculated

☐ Tick this box to confirm that the raw and calibrated dates are available in the paper or in Supplementary Information.

Ethics oversight	no ethical oversight was required, as the study only used museum collection material

Note that full information on the approval of the study protocol must also be provided in the manuscript.

Plants

Seed stocks	no plants involved in this study
Novel plant genotypes	n/a
Authentication	n/a
